# Giant lung and liver hydatid cyst in a 3-year-old child

**DOI:** 10.1590/0037-8682-0175-2022

**Published:** 2022-09-19

**Authors:** Mesut Ozgokce, Kemal Ayengin, Yener Aydin

**Affiliations:** 1 Yuzuncu Yil University, Medical Faculty, Department of Radiology, Van, Turkey.; 2 Yuzuncu Yil University, Medical Faculty, Department of Pediatric Surgery, Van, Turkey.; 3 Ataturk University, Medical Faculty, Department of Thoracic Surgery, Erzurum, Turkey.

**FIGURE 1:**
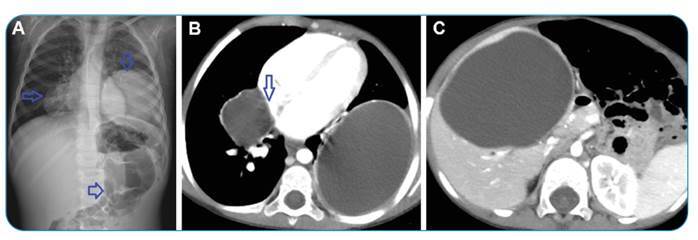
Direct radiography shows a radiopaque appearance in the right lower zone of the lung in the paracardial region and the left lower zone of the lung. The homogeneous opacity increase (arrows) pushed on the intestinal loops **(A)**. An axial thoracic computed tomography **(CT)** mediastinal section shows a cyst in the right middle lobe **(arrow)** compressing the right atrium and a giant hydatid cyst filling the lower lobe of the left lung **(B)**. An abdominal CT section shows a giant hydatid cyst with similar characteristics in the liver **(C)**.

A 3-year-old girl presented with growth retardation. Radiologically, a radiopacity consistent with a hydatid cyst was noted in both the lungs and the liver (Figure 1). The patient underwent surgical treatment to remove the cyst from the lungs and liver. 

Hydatid disease is a parasitic disease transmitted via the fecal-oral route1. In approximately 8% of cases, the hydatid cyst is located in both the liver and lungs^1^. When the hydatid cyst is intact, it appears on direct radiographs as a round radiopacity with smooth margins. In endemic areas, the diagnosis is made by clinical and radiologic findings. Growth retardation rarely occurs in hydatid cysts^1^. Serology may help diagnose, especially if the cyst ruptures and the case becomes complicated^2^. Although hydatid cysts can be large, the coexistence of giant cysts in both the liver and lungs is rare in 3-year-old children^3^. In these cases, the hydatid cyst should be treated immediately.
